# Supplementary material for: Neuropathic pain in patients with osteoarthritis of the hip before and after total hip arthroplasty
Source: PLoS One. 2024 Apr 25;19(4):e0301352. doi: 10.1371/journal.pone.0301352 (PMC11045094; doi:10.1371/journal.pone.0301352)
Supplement: Attachment — Submitted filename: Response to Reviewers.docx [file pone.0301352.s002.docx]

Responses to the comments by the Associate editor:

1. **Please ensure that your manuscript meets PLOS ONE's style requirements, including those for file naming.**

[Response]

Thank you for your comment. We have confirmed that our manuscript fits the PLOS ONE’s style.

2. **During our internal evaluation of the manuscript, we found significant text overlap between your submission and the following previously published works, some of which you are an author.** **Please revise the manuscript to rephrase the duplicated text and fully cite all your sources, where appropriate.**

[Response]

Thank you for your comment. I have revised the manuscript overlapping based on your suggestion as below. Please let me know if other areas need to be corrected.

(Page 3, Lines 27; Page 4, Lines 69-73; Page 4, Lines 78-100)

3. **We note that your Data Availability Statement is currently as follows: “All relevant data are within the manuscript and its Supporting Information files.”Please confirm at this time whether or not your submission contains all raw data required to replicate the results of your study.**

[Response]

We have confirmed the above.

4. **Your ethics statement should only appear in the Methods section of your manuscript. If your ethics statement is written in any section besides the Methods, please move it to the Methods section and delete it from any other section. Please ensure that your ethics statement is included in your manuscript, as the ethics statement entered into the online submission form will not be published alongside your manuscript.**

[Response]

Thank you for your helpful recommendation. We have moved the ethics statement to the Methods section as below. (Page 4, Lines 71)

“This study was approved by the institutional review board of Mie University Hospital (H2018-083) and all patients received written informed consent to participate.”

Responses to the Comments by the reviewer 1:

**・As the author described, there were some tools to assess the neuropathic pain. I think that the author should perform another assessment.**

[Response]

Thank you for your valuable suggestions. We understand that we should perform another assessment. The main reason why we chose the PDQ is that we have a Japanese-language version of this screening tool. Because of the language barrier, it isn't easy to use another self-reporting screening tool in our country, especially for elderly patients. In addition, we compared the usefulness of each tool based on the references (5-6,16 in the reference list) and determined that the PDQ is a simple and reliable tool. Unfortunately, it is impossible to attempt another assessment on the same patients from now because of the characteristics of this study. We added the following sentence as a limitation. (Page 9, Lines 176)

“We have used only PDQ and NRS to verify the association between the presence of preoperative neuropathic pain and the occurrence of chronic postoperative pain.”

**・Was the timing of the assessment the same for all patients? The author should describe the timing, who assessed, whether NRS on rest or activity, and whether medication such as pain killer was performed or not.**

[Response]

Thank you for your great comments. According to your suggestion, we have fixed and added the sentences as below.

(Page 4, Lines 67)

“The PDQ was administered preoperatively and 6 months postoperatively in all patients, and the prevalence of neuropathic pain was measured before and after THA by the surgeon. Sex, age, BMI, and numerical rating scale (NRS) scores on rest were compared between groups.”

(Page 4, Lines 54)

“For the first two weeks after surgery, all patients took oral NSAIDs or acetaminophen according to our protocol. No patients used analgesics specifically for neuropathic pain.”

**・The patients who had spine disorders should be investigated.**

[Response]

Thank you very much for your excellent comments. We also determined that spine disorders needed to be ruled out, and we examined all patients as much as possible preoperatively. Specifically, all patients underwent an interview and spinal X-ray to detect lumbar spine disease. In addition, a lumbar spine MRI was performed on those patients who seemed to need it. Patients whose hip pain is suspected of neuropathic pain because of spinal disorders were not indicated for THA. According to your suggestion, we have added the following sentences to the Methods section and Discussion section as below.

(Page 4, Lines 52)

“Patients whose preoperative physical and imaging findings suggested that their hip pain was due to spine-related disease were not indicated for THA.”

(Page8, Lines 159)

“In this study, all patients underwent an interview and X-ray to detect lumbar spine disease. In addition, a lumbar spine MRI was performed on those patients who seemed to need it. It is difficult to completely rule out the involvement of lumbar spine disease preoperatively and elderly patients with neuropathic pain should be searched more carefully for spinal disease before THA”

**・The surgical approach was described to affect postoperative pain. The surgical approach should be united when the pain study.**

[Response]

Thank you for your comment. As you indicated, the surgical approaches should have been unified. Since the various approaches were mixed in the following references (16-19 in the reference list), we did not unite the approaches in this study. We added the following sentence as a limitation. (Page 9, Lines 179)

“Surgical approaches have not been unified, and the effect of different approaches on neuropathic pain has not been studied.”

**・The evidence to prove the association between the presence of preoperative neuropathic pain and the occurrence of chronic postoperative pain after THA was insufficient.**

[Response]

Thank you for your helpful recommendations. In our research, a significantly higher postoperative NRS score was observed in the preoperative NP group (0.76 ± 1.25) than in the preoperative non-NP group (0.30 ± 0.78) after THA. In addition, a positive correlation was observed between the preoperative PDQ and postoperative NRS scores (r = 0.375, p < 0.001). While there may not be a large difference in the calculated scores, for these reasons we determined that there is an association between the presence of preoperative neuropathic pain and the occurrence of chronic postoperative pain after THA. We added the following sentence as a limitation. (Page 9, Lines 176)

“We have used only PDQ and NRS to verify the association between the presence of preoperative neuropathic pain and the occurrence of chronic postoperative pain.”

**Line 61: I think including “unclear pain” into NP group was not appropriate.**

**Line 72: As I wrote above, the definition of NP group and non-NP group was not appropriate. The authors should assess the correlations between the score of PDQ and NRS score and BMI.**

[Response]

Thank you for your comment. We consider this grouping is appropriate because all of the cited references (16-19 in the reference list) that evaluated neuropathic pain using the PDQ set the cutoff value at 13 and included unclear pain into the NP group. Since we have already discussed the correlation between PDQ and NRS, we have added an examination of the correlation between PDQ and BMI to the methods and discussion as below.

(Page 4, Lines 70)

“The correlation between the preoperative PDQ score and the pre- and postoperative NRS scores and between the preoperative PDQ score and BMI were also investigated.”

(Page 4, Lines 80)

“Spearman's correlation coefficient was used to determine the correlations between pre- and post-THA NRS scores and preoperative PDQ scores, and between BMI and preoperative PDQ scores.”

(Page 5, Lines 117)

“A correlation was not observed between the preoperative PDQ and BMI (r = 0.052, p = 0.51).”

**Line 89: Please show average and standard deviation or standard error on NRS score, Age and BMI.**

[Response]

Thank you for providing these insights. According to your suggestion, we have changed the notation of data as the average and standard deviation on NRS score, age and BMI in Table2 (Page6, Lines 130).

Responses to the Comments by the reviewer 2:

**VERY INTERESTING TOPIC.
HOWEVER, I BELIEVE THAT THE LACK OF DATA RELATING TO THE COEXISTENCE OF SPINE DISEASES CAN DETERMINE A BIAS. IN THIS REGARD, HOW DO YOU EXPLAIN THE CORRELATION BETWEEN ADVANCED AGE AND GREATER INCIDENCE OF NEUROPATHIC PAIN? TO OPTIMIZE THE RESULTS IT IS ESSENTIAL THAT AT LEAST IN THIS GROUP OF PATIENTS THE COEXISTENCE OF VERTEBRAL PROBLEMS IS EXCLUDED.**

[Response]

Thank you very much for your excellent comments. We also determined that spine disorders needed to be ruled out, and we examined all patients as much as possible preoperatively. Specifically, all patients underwent an interview and spinal X-ray to detect lumbar spine disease. In addition, a lumbar spine MRI was performed on those patients who seemed to need it. Patients whose hip pain is suspected of neuropathic pain because of spinal disorders were not indicated for THA. According to your suggestion, we have added the following sentences to the Methods section and Discussion section as limitation.

(Page 4, Lines 52)

“Patients whose preoperative physical and imaging findings suggested that their hip pain was due to spine-related disease were not indicated for THA.”

(Page8, Lines 159)

“In this study, all patients underwent an interview and X-ray to detect lumbar spine disease. In addition, a lumbar spine MRI was performed on those patients who seemed to need it. It is difficult to completely rule out the involvement of lumbar spine disease preoperatively and elderly patients with neuropathic pain should be searched more carefully for spinal disease before THA”

(Page9, Lines 175)

“A correlation between age and the incidence of neuropathic pain may have occurred because spinal disease could not be completely ruled out.”
